# Perceived benefits and limitations of a psychoeducation program for patients with fibromyalgia: an interpretative phenomenological analysis

**DOI:** 10.3389/fpsyg.2024.1422894

**Published:** 2024-08-14

**Authors:** Silvia Di-Bonaventura, Raúl Ferrer-Peña, Joaquín Pardo-Montero, Josué Férnandez-Carnero, Roy La Touche

**Affiliations:** ^1^Escuela Internacional de Doctorado, Department of Physical Therapy, Occupational Therapy, Rehabilitation and Physical Medicine, Rey Juan Carlos University, Alcorcón, Spain; ^2^Department of Physical Therapy, Occupational Therapy, Rehabilitation and Physical Medicine, Rey Juan Carlos University, Alcorcón, Spain; ^3^Cognitive Neuroscience, Pain and Rehabilitation Research Group (NECODOR), Faculty of Health Sciences, Rey Juan Carlos University, Madrid, Spain; ^4^Clinico-Educational Research Group on Rehabilitation Sciences (INDOCLIN), CSEU La Salle, Universidad Autonóma de Madrid, Madrid, Spain; ^5^Department of Physiotherapy, Faculty of Health Sciences, CSEU La Salle, Universidad Autonóma de Madrid, Madrid, Spain; ^6^Hospital La Paz Institute for Health Research (IdiPAZ), Madrid, Spain; ^7^Motion in Brains Research Group, Centro Superior de Estudios Universitarios La Salle, Universidad Autónoma de Madrid, Madrid, Spain; ^8^Musculoskeletal Pain and Motor Control Research Group, Faculty of Sport Sciences, Universidad Europea de Madrid, Villaviciosa de Odón, Spain; ^9^Instituto de Dolor Craneofacial y Neuromusculoesquelético (INDCRAN), Madrid, Spain

**Keywords:** fibromyalgia, psychoeducation, pain management, non-pharmacological treatment, interpretative phenomenological analysis

## Abstract

**Objective:**

To analyze the perceived benefits and limitations of a pain psychoeducation program as a non-pharmacological treatment for patients with fibromyalgia.

**Methods:**

An interpretative phenomenological analysis was applied to analyze the subjective experiences of 11 patients with fibromyalgia who participated in a pain psychoeducation program. This program includes educational sessions that address pain understanding, coping strategies, and relaxation techniques. Semi-structured interviews were conducted, transcribed, and analyzed using ATLAS.ti software.

**Results:**

Patients reported significant improvements in cognitive-functional capacity and socio-emotional ability, including better disease understanding and management, emotional stability, and interpersonal relationships. Despite these benefits, they identified limitations in program individualization and insufficient coverage of certain topics, such as sexual health and legal aspects of disability. Enhanced self-management skills were evident, with observed shifts in disease perception and coping strategies.

**Conclusion:**

The psychoeducation program was viewed positively, influencing functional, cognitive, and emotional enhancements. Nonetheless, the need for increased program personalization and expanded socio-economic support was noted. Future research should focus on the long-term impacts of psychoeducation and the feasibility of tailored interventions.

## Background

Fibromyalgia disease, a chronic pain condition whose prevalence continues to increase steadily (Nahin et al., 2023), represents a major challenge for sufferers and healthcare professionals (Polacek et al., 2020; Kachaner et al., 2023; Sturgeon et al., 2024). This condition is a particular clinical entity, characterized by the presence of widespread pain, as well as several associated symptoms that can vary in intensity and duration (Choy et al., 2010; Silverman et al., 2010; Horta-Baas and Romero-Figueroa, 2019). Patients coping with this condition must not only deal with the physical pain burden (Schaefer et al., 2011; Perrot, 2012), but also with functional limitations, socioeconomic consequences, and a diminished quality of life (Fernandez-Feijoo et al., 2022). Despite the prevalence of fibromyalgia and its impact on society, the diagnosis and management of this condition remain major challenges (Choy et al., 2010; Qureshi et al., 2021; Van Wilgen et al., 2023). In this context, pharmacological treatment has been a conventional strategy in the approach to fibromyalgia. However, the medications used to treat different symptoms of this condition present notable limitations in terms of efficacy (da Rocha et al., 2019; Redondo et al., 2022), and many are accompanied by adverse effects (Crofford, 2010; Tzellos et al., 2010). In addition, the issue of pharmacological treatments’ cost-effectiveness has raised concerns in an ever-changing health care context (Rathore and Afridi, 2020). Therefore, there is an increasing need to explore non-pharmacological avenues that consider the biopsychosocial model for fibromyalgia treatment.

Traditionally, qualitative approaches, such as interpretative phenomenological analysis (IPA), it has been used to understand patients’ beliefs and thoughts in more detail (Smith, 1996; Tindall, 2009). This qualitative methodology allows healthcare professionals to examine the complex interplay between the biological, psychological, and social aspects of chronic pain (Smith, 1996). In this context, pain education has emerged as a promising strategy. Pain education focuses on empowering patients by providing them with information, skills, and tools to understand and manage their pain effectively (Barrenengoa-Cuadra et al., 2021). This approach goes beyond simply informing patients about their condition; it provides patients with the tools to better understand the complexities of chronic pain, encourages active and effective coping, and promotes self-management of symptoms (Louw et al., 2016; Areso-Bóveda et al., 2022). The incorporation of multidisciplinary approaches in the treatment of chronic pain, particularly in fibromyalgia, is key to effective management. Therapeutic education serves as a bridge to these approaches, helping make treatment more effective. It allows collaboration between different specialties, such as psychology, physiotherapy, and pain medicine, offering a comprehensive strategy that encompasses both pain relief and the overall well-being of the patient. This multidisciplinary approach ensures that all aspects of chronic pain are addressed, from the physical to the psychosocial, promoting a more complete and personalized treatment (Kamper et al., 2015). In this manner, classical cognitive-behavioral therapy (CBT) has become a key component in managing patients with fibromyalgia (Heller et al., 2021), guiding patients toward a better relationship with their pain, incorporating it as an integral part of their lives, and discouraging any excessive attempts to control or avoid it (Dindo et al., 2017). Here, acceptance should not be mistaken for resignation; rather, it is a conscious acknowledgment of the presence of pain and a firm resolution to move forward despite it. This facilitates an improvement in patients’ quality of life, encouraging them to engage in meaningful activities and maintain active social relationships, even in the face of limitations imposed by chronic pain (Hughes et al., 2017). However, despite the potential benefits mentioned, there remains a significant gap in knowledge regarding how to effectively implement these psychoeducational strategies in the management of fibromyalgia. The aim of this article is to critically examine perceptions of the benefits and limitations of pain psychoeducation as a non-pharmacological treatment in patients with fibromyalgia.

### Study design

This study was designed in accordance with the Consolidated Criteria for Reporting Qualitative Research (Tong et al., 2007) and the Standards for Reporting Qualitative Research (O’Brien et al., 2014).

A cross-sectional qualitative methodology was developed through IPA as described by Smith (1996). This is a flexible and versatile qualitative method of analysis that assesses the significance individuals attribute to their lived experiences (Pringle et al., 2011; Tuffour, 2017; Duque and Díaz-Granados, 2019). It is characterized by its approach to explore, describe, interpret, and contextualize the interpretations of experiences (Larkin et al., 2006, 2019) from a small number of participants (Tindall, 2009). IPA was specifically chosen for its ability to delve deeply into complex perceptions and experiences associated with persistent pain, enabling researchers to gain detailed and subjective insights that are crucial for a better understanding of a psychoeducation program from the patients’ perspective.

### Ethics

The study was approved by the Bioethics Committee of the Rey Juan Carlos University (code: 1505202323023). It was conducted in accordance with the Ethical Principles of the World Medical Association (Helsinki Declaration).

Prior to conducting the semi-structured interview, participants voluntarily signed a written informed consent that included their agreement to participate in the study, permission to audio record the interview, and a series of clauses related to confidentiality, in which the researcher committed to not disclosing any personally identifiable information, thereby ensuring the anonymity of the participants at all stages of the study analysis. A code in both numerical and letter format was assigned to each participant.

### Research team

During three meetings, the research team discussed various scientific aspects related to psychoeducation in the management of patients with chronic pain. The theoretical framework, the motivation for conducting the research, and the positioning of each researcher were defined in advance of the study’s execution (O’Brien et al., 2014).

The research team for this study was comprised of physiotherapists and psychologists with extensive experience in both research and clinical treatment of patients with pain.

The structured questions for the interview were developed by RLT, who holds a Ph.D. in pain research, and JPM, a clinical psychologist with a Ph.D. and more than 25 years of clinical experience in patients with pain. The interviewer responsible for conducting the interviews, who underwent prior training in qualitative interviewing skills, was SDB, a doctoral candidate in pain research, holding a related master’s degree and over 5 years of experience working with patients experiencing persistent pain.

Before the commencement of the study, the interview questions were validated with experts. There was no pre-existing relationship between the participants and the researchers RLT, JPM, and SDB. The latter was introduced to the participants, explaining her role and the motivation behind conducting the research, which is aimed at a better understanding of their perceptions of the fibromyalgia patient education program. The entire research process was supervised by RFP and JFC, both of whom have a Ph.D. in pain research.

### Context

The study was conducted at the Fibromyalgia and Chronic Fatigue Association of Mostoles in Madrid. This association provides information and advice, as well as organizing activities and individualized therapy for patients with fibromyalgia and chronic fatigue. However, the interviews and data collection were conducted in a private office within the premises of Rey Juan Carlos University. During the interview sessions, only the researcher and the participant were present, with no third parties involved, to ensure a trustworthy and confidential environment that facilitated openness and honesty in the participants’ responses.

### Participants

Inclusion criteria encompassed adults diagnosed with fibromyalgia who had been part of the psychoeducation group for 9 months and who had the ability to complete questionnaires and the interview (Table 1).

**TABLE 1:** Socio-demographic variables.

ID	M/F (male/female)	Age (years)	Pain duration (years)	BMI	Marital status	Job status	Education
1	F	63	20	32.5	Married	Retired	Tertiary
2	F	61	11	19.6	Married	Homemaker	Upper secondary
3	F	60	36	27.4	Married	Homemaker	Upper secondary
4	F	56	30	33	Married	Sick leave	Tertiary
5	F	62	16	27.7	Divorced	Sick leave	Tertiary
6	F	33	7	26.4	Married	Unemployed	Upper secondary
7	F	50	13	32.5	Single	Student	Tertiary
8	M	49	7	25.4	Married	Sick leave	Primary
9	F	57	6	23	Married	Unemployed	Upper secondary
10	F	58	18	26.9	Married	Homemaker	Primary
11	F	63	50	27.1	Married	Homemaker	Tertiary

In addition to the previously outlined inclusion criteria, participants were also selected based on their availability and willingness to share their experiences related to the psychoeducation program. Initial contact with the association was made via telephone by the researcher JFC, and information about the study was distributed in writing via email. The association disseminated informational leaflets about the study among its members, providing details on the research objectives and how interested parties could register to participate. This method allowed patients affiliated with the association who were interested in sharing their experiences with fibromyalgia to voluntarily enroll in the study.

### Psycho-education program

**FIGURE 1:**
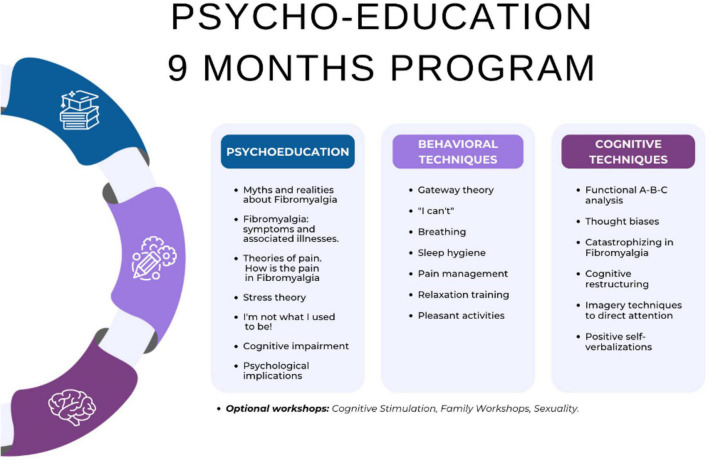
Psycho-education program content.

The psychoeducation program consisted of weekly 1-h sessions, organized into three quarterly modules, each comprising 12 sessions (Figure 1). The intervention mode was mixed, combining both in-person and remote attendance, with an average of 7 to 9 patients attending each session. Upon completing the three mandatory psychoeducation modules, patients had the option to participate in additional workshops focusing on self-esteem and social skills, which were also offered over 3-month periods with 12 sessions each. Progression to subsequent modules, both in the psychoeducation program and the additional workshops, required a minimum attendance of 80%. Those who did not meet this requirement had to repeat the module. In our study, all patients had completed the three modules of psychoeducation. To avoid biases in the evaluation of the program’s results, the patients included in this study had not begun the optional sessions at the time of evaluation.

### Sampling strategy

Purposive sampling was employed for the selection of study participants, a method that is based on choosing potential participants according to the research question and the objectives of the study (Teddlie and Yu, 2007).

The sample size comprised 11 patients (10 women and 1 man), mirroring the actual proportion of individuals affected by fibromyalgia (Buskila et al., 2001). Only one individual who initially agreed to participate ultimately could not do so due to an unforeseen medical appointment. No dropouts were recorded during the study. The selected sample size for this research aligns with previous recommendations suggesting the inclusion of 2 to 12 participants for conducting an interpretive phenomenological analysis (Noon, 2018).

### Data collection

Semi-structured in-depth interviews were conducted using an interview guide developed based on the research team meetings and scientific evidence related to psychoeducation in patients with pain (Supplementary Annex 1). The interview procedure was designed according to the criteria outlined by DeJonckheere and Vaughn (2019).

The interview questions were pre-tested on a pilot group of patients with fibromyalgia who did not participate in the final study, allowing for adjustments and enhancements to the interview script. Thanks to this preliminary test, it was not necessary to conduct repeat interviews.

Audio recording devices were utilized to capture the interviews, with all participants being duly informed beforehand and providing their consent for such recordings. The interviews varied in length, ranging from 29 to 55 min, with an average duration of approximately 42 min. The audio-recorded interviews were transcribed verbatim.

To elicit more comprehensive responses or when participants provided inadequate or terse answers, follow-up strategies were implemented. These included probing questions, such as *“Could you please elaborate on that?”*, *“What do you mean by that?*”, or “*Could you tell me more about…?*” These supplementary questions were flexibly applied to achieve a more in-depth understanding of the patients’ experiences and viewpoints.

SDB took field notes during the interviews to ensure thorough coverage of the topics discussed. Directly following each interview, reflective notes were made regarding the significance of the discussion themes to facilitate the analysis. These notes were also referenced during the transcription and analysis phases to incorporate reflective commentary on the researcher’s insights and the interpretative process. Although the interview transcripts were not automatically returned to the participants for comments or corrections, they were informed that they had the option to request and review their transcripts if they wished.

### Variables

• Anxiety and depression were assessed using the Spanish-validated version of the self-completed Hospital Anxiety and Depression Scale (HADS). This scale is divided into two subscales of seven items each: 1) depression (HADSDep); and 2) anxiety (HADSAnx). The HADS has demonstrated good reliability and validity in various pathologies. The cutoff points used to assess the presence and severity of symptoms are as follows: 0–7 for no anxiety or depression (normal), 8–10 for mild anxiety or depression (borderline cases), and 11–21 for moderate to severe anxiety or depression (clinical cases) (Bjelland et al., 2002).
• The Chronic Pain Grading Scale (CPGS) is a self-report instrument consisting of an eight-item scale. It has a high internal consistency, with a Cronbach’s α of 0.87, similar to that of other language versions, and an intraclass correlation coefficient of 0.81. The average administration time is 2 min 28 s (Von Korff et al., 1992). For this instrument, we used two cutoff points to classify the patients: Grade I, moderately limiting (<24 points); Grade II, severely limiting (>25 points).
• The Pain Self-Efficacy Questionnaire (PSEQ) is a self-administered instrument designed to assess self-efficacy in individuals with chronic pain. This scale consists of 10 items that measure an individual’s confidence in performing daily activities despite pain. The PSEQ has shown high internal consistency, with a Cronbach’s alpha of 0.92, which is consistent with versions in other languages, and an intraclass correlation coefficient of 0.89, indicating good test-retest reliability. The average administration time is approximately 3 min (Dubé et al., 2021).
• Global Perception of Change is the only variable tested after the intervention. This instrument consists of a 100-mm line numbered in centimeters from −5 to 5, in which the left end reads “much worse,” 0 at the center is “no change,” and 5 at the right end is “fully recovered.” Relative to these values, the patient is asked to answer the following question: “In relation to your pain, how would you describe your current state of health compared to when your pain began?” This version of the change perception scale has been assessed in the literature as the most appropriate in musculoskeletal pain processes, and as a valid and reliable instrument (Kamper et al., 2009).
• Pain intensity was measured with a 100-mm numeric rating scale (NRS), in which 0 represents “no pain” and 100 the “worst pain imaginable.” Participants draw a mark at a point on the line that best reflects the pain they are experiencing at the time of measurement. Higher scores indicate higher pain levels. The sensitivity and specificity of this questionnaire and the acceptability of its psychometric properties have been approved (Jensen and McFarland, 1993).
• The biobehavioral pain and movement questionnaire (BioPMovQ) is a measure for motor and functional impairments due to pain, with a standard error of measurement (SEM) of 3.43, in which higher scores denote more severe disturbances, and with cut-off points at ≥37 for moderate (sensitivity: 0.87, specificity: 0.59) and ≥45 for severe impairment (sensitivity: 0.72, specificity: 0.98) (La Touche et al., 2024).

### Data analysis

**FIGURE 2:**
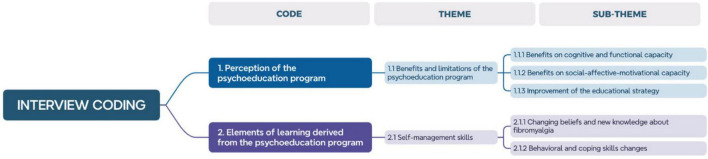
Interview coding.

The data coding process was performed in three rounds to ensure an accurate and consistent interpretation. The first two rounds were conducted by two experienced qualitative data coders, in direct collaboration with the researchers (RFP and RLT). During these rounds, they worked on defining and adjusting the coding tree, seeking consensus and a clear understanding of the data. The third and final round of coding also involved the researcher who conducted the interviews, SDB, with the aim of incorporating an additional perspective based on direct knowledge of the responses and experiences shared by the participants during the interviews. The detailed structure of the coding tree, as well as its development, are described in the research report (Figure 2). Regarding data saturation, a point was reached at which no new themes or significant ideas emerged, indicating that data collection had been sufficient to capture the relevant experiences of the participants.

Themes were identified inductively from the data, allowing a genuine and grounded interpretation of the participants’ experiences. ATLAS.ti software (Soratto et al., 2020) was used for data management and analysis.

### Reporting

We presented participants’ quotes to illustrate the findings and to identify themes. Each quote was associated with a participant number, thus maintaining confidentiality and providing a clear context for each statement. This approach allowed a direct link between the personal experiences of the participants and the emerging themes from the analysis.

The structure of the coding tree was divided into codes, themes, and subthemes, facilitating a clear organization and accurate interpretation of the data. This division allowed a detailed representation of the findings and ensured that the data presentation was aligned with the study’s conclusions. The main themes were highlighted in the findings, providing a clear view of the most relevant and recurring aspects in the participants’ experiences. These central themes were detailed, leading to a thorough understanding of the critical points of the study. Additionally, the diversity of cases was addressed, and secondary or minor themes were discussed, reflecting the variety of experiences within the sample. This attention to minor themes added depth and nuance to the study, resulting in a complete and more diverse picture of the experiences related to fibromyalgia.

## Results

A descriptive analysis of the data suggests that patients experience pain at a considerable level, ranging from moderate to severe on the NRS scale, indicating significant pain intensity. Despite this, the Global Rating of Change (GROC) score reflects a general perception of improvement, suggesting that patients recognize progress in their condition, which can be considered as favorable within the parameters of this scale. As for the PSEQ, scores were at the upper end of the range, implying high self-efficacy in pain management. However, the levels of anxiety and depression, as measured by the HADS, are in a range that indicates the presence of psychologically relevant symptoms. Lastly, the classification of chronic pain, in which the majority is Grade II according to the CPGS, and the scores of the BioPMovQ survey, reveal a considerable impact on patients’ functionality and quality of life, requiring attention toward comprehensive pain management.

The general perception of the psychoeducation program among participants is summarized in two main codes emerging from the interviews: *Perception of the Psychoeducation Program* and *Elements of Learning Derived from the Psychoeducation Program*. These codes reflect an extremely positive valuation of the program, demonstrating how it significantly improved their life outlook and provided them with useful tools and knowledge to face their daily challenges in a more effective and healthy manner, which can be evidenced in the high satisfaction levels presented in the GROC satisfaction scale (Table 2).

**TABLE 2:** Psychosocial variables measured at the end of the program.

ID	NRS	GROC	PSEQ	HADS anxiety	HADS depression	GCPS total	GCPS intensity	GCPS disability	GCPS grade	BioPMovQ
	[0 to 100]	[−7 to 7]	[0 to 190]	[0 to 21]	[0 to 21]	[0 to 70]	[0 to 30]	[0 to 40]	[I to II]	[0 to 64]
1	80	3	71	9	10	54	23	31	II	53
2	70	7	131	9	7	36	24	12	I	32
3	80	4	80	12	9	53	23	30	II	50
4	55	5	183	5	2	24	18	6	I	26
5	70	6	100	7	8	54	23	31	II	45
6	70	−4	34	14	12	57	24	33	II	50
7	70	5	154	10	6	33	22	11	I	39
8	35	7	60	15	17	53	22	31	II	61
9	60	6	102	9	3	48	22	26	II	51
10	80	4	101	15	10	51	24	27	II	50
11	60	6	104	8	4	52	23	29	II	36
Mean	66.4	4.5	101.8	10.3	8	46.8	22.5	24.3	–	44.8
SD	13.4	3.1	42.4	3.3	4.3	10.8	1.7	9.7	–	10.5

ID, identification; NRS, numeric rating scale; GROC, global rating of change scale; PSEQ, pain self-efficacy questionnaire; HADS, hospital anxiety and depression scale; GCPS, graded chronic pain scale (Grade I, moderately limiting; Grade II, severely limiting); BioPMovQ, biobehavioral pain and movement questionnaire.

The first code, *Perception of the Psychoeducation Program*, highlights not only the positive reception of the program by the participants, but also how they have experienced improvements in various aspects of their life thanks to this. Likewise, emphasis is placed on the limitations they wish to highlight, focusing on those aspects that often go unnoticed but are crucial for optimizing the effectiveness of the pain management program and the patients’ general well-being.

The second code, *Elements of Learning Derived from the Psychoeducation Program*, describes the transformative impact of the program on the participants, especially in how it has provided them with more tools to face a condition like fibromyalgia. This code highlights how psychoeducation has facilitated a deeper understanding of their illness, providing strategies to better manage the symptoms and, consequently, improve their quality of life.

Both codes encompass specific themes and subthemes that will be detailed below.

### Benefits and limitations of the psychoeducation program

Within the theme Benefits and Limitations of the Psychoeducation Program, several key subthemes emerged that demonstrate how this program has positively influenced participants, while also pointing out areas for improvement. The observed benefits were distributed across three main areas: improvements in functional cognitive capacity, encompassing an increase in understanding and management of the condition; benefits in emotional, affective, and social capacity, reflecting how participants have achieved greater emotional stability and better interpersonal relationships despite perceived difficulties; and lastly, the limitations of the program, in which participants suggest adjustments to maximize its impact, especially in terms of aspects that are often less considered but equally important for holistic well-being. To encapsulate the general perception of participants toward the program, we highlight the following statements, illustrating the significant impact on their lives:

*“It improves your life, in all aspects.”*

*“For me, it has certainly been fortunate to find something like this.”*

*“It was, is, and will be very important for my entire fibromyalgic life. I always recommend it whenever I can.”*

#### Benefits for functional-cognitive capacity

Participants reported notable benefits in the psychoeducation program in terms of their functional and cognitive capacity, especially in the improvement of physical activities and key cognitive aspects such as memory and concentration, commonly affected by fibromyalgia. Testimonies such as “Before, I couldn’t walk as much as I can now; I almost walk for an hour and I don’t get tired!” reflect an increase in the capacity to perform exercise, attributed to the program’s recommendation to integrate regular physical activity, which had a positive effect on pain reduction and on the improvement of general well-being. Experiences also highlight how the program influenced the improvement of concentration and pain management, with participants expressing that *“The program has helped me, it has connected me, it has instilled in me to do physical exercise… and it has indeed been beneficial*” and “*It has helped me to concentrate a bit more because I couldn’t concentrate on anything.*” Moreover, a greater capacity for attention in daily life was reported, overcoming previous states of distraction, as reflected in comments such as *“They have made me see that now I am more focused, more attentive; because before, I was absent.”*

#### Benefits on emotional-social-affective capacity

Lastly, the impact on the emotional-affective capacity was perhaps the most significant. Learning to manage emotions and see the positive in difficult situations is crucial for mental health and emotional well-being. One participant put it this way: “*They have taught us to see the positive, to change our focus to live better with it. That is, to live with these limitations and the difficulties that come with fibromyalgia, but understanding that you can always fight, improve, or make the most of the good moments.*” Many participants developed this positive approach, attributed in large part to the supportive group environment created by the program, which they considered essential for fostering motivation and a resilient spirit in the face of adversity. This collective support was vividly reflected in their expressions, as when one of the participants says: “*You will see that you are not alone, that you have a group that is going through the same as you and supports you*,” underscoring the sense of community and mutual encouragement that was fostered among the participants.

#### Improvements of the educational strategy

Participants in the program identified key areas requiring improvements to increase the impact and efficacy of the treatment offered. A primary concern centered on performing exercises at home, where the lack of direct supervision generated doubts about correct execution and reduces adherence to these routines. Statements such as “*What I find most difficult is doing the exercises at home because I only think that maybe I’m doing them wrong and sometimes I end up not doing them*” illustrates the anxiety some patients feel when trying to follow the routines on their own, demonstrating the need for clearer instructions or more personalized follow-up. Additionally, there was a clear request for specialized workshops addressing less discussed but equally important topics, such as sexuality, which some patients believe is affected by the physical limitations and pain associated with fibromyalgia. This need underscored the importance of considering sexual health as a critical component of the quality of life for people with this condition. Patients have also expressed a need for guidance on managing legal and disability issues, reflecting the desire for a more comprehensive treatment approach that includes legal and specialized support from the beginning. “*I think we should have more support from lawyers, experts, etc., because for us, this is something unknown and also very costly as a service*” said a participant, highlighting the importance of having a multidisciplinary team. Lastly, patients stress the importance of having more individualized treatment options tailored to their specific needs: *“The problem is time. If you want to go deeper, you would have to take and do something a bit more individualized, not always in a group*,” another participant said, showing the need for a more personalized approach.

### Self-management skills

The psychoeducation program had a transformative impact on participants by fostering the development of self-management skills in dealing with fibromyalgia, addressing everything from changing erroneous beliefs about the disease and acquiring new knowledge, to learning cognitive-emotional coping skills and implementing behavioral modifications. These testimonies reflect an evolution in the understanding and management of fibromyalgia; participants have moved from viewing it as a degenerative and limiting illness to recognizing that, with adequate information and management strategies, it is possible to lead an active and fulfilling life. They learned that pain and cognitive scatter are manageable, and that the disease does not necessarily lead to total disability. Through the program, participants discovered the importance of physical exercise, even if adapted, and have integrated these activities into their daily routine, evidencing a significant change in their approach to pain management and their ability to perform everyday activities. The customization of exercise and its gradual implementation have become key to their success. Moreover, the adoption of relaxation techniques and acceptance of the emotional ups and downs characteristic of fibromyalgia have improved their emotional well-being, allowing them to face the challenges of the disease with a new perspective.

#### Changing beliefs and new knowledge about fibromyalgia

The psychoeducation program has been fundamental for redefining perceptions and expanding participants’ knowledge about fibromyalgia, transforming their previous beliefs and offering them a deeper understanding of their condition. Previously, many harbored the idea that they were facing a degenerative disease, with fears of memory loss and a life of disability. However, the program has revealed to them that “*The only belief that was very helpful to me was that I thought this was a degenerative disease, and here they explained to me that it was not degenerative and that it’s not really about losing memory, but that the pain makes you distracted*.” This new perspective has been enlightening, changing the vision of the disease from one of limitation to one of management and adaptation. Moreover, participants have discovered unknown aspects of fibromyalgia, learning about its causes, effects, and how to mitigate its symptoms. This education has been vital, as reflected in the experience of a participant: “*I have learned many things that I was completely unaware of… As a result of that, I have had a very important improvement.*” By sharing these experiences and knowledge, a sense of community and mutual support has been fostered, strengthening individual and collective empowerment against the disease. “*Here I learned that fibromyalgia does not disable you*”, and “*They explained to me that it’s not a disease that you’re going to die from*” summarize the change of perspective experienced by the participants, marking a “before and after” in their way of living with fibromyalgia.

#### Behavioral and coping skills strategies

The psychoeducation program had a profound impact on the development of cognitive-emotional coping skills and on the implementation of behavioral modifications in participants, teaching them to live adaptively with fibromyalgia. Through it, they learned that confronting the disease with a proactive and positive attitude is essential, as reflected in the statement, “*It has been fundamental to learn how to fight against this, to live better and not in opposition; because it’s something we have and it will go with us, but that doesn’t mean it has to cut our life short*.” This philosophy of life extends to pain management, in which distraction and acceptance of emotional ups and downs are key: *“The more you ignore the pain or the more you distract yourself from it, the better you can carry out your activities… I have learned to adapt and continue fighting. And I have changed the way I see life.*” Behavioral modifications, such as incorporating regular physical exercise and adapting daily activities to their capabilities, have been equally transformative. The transition from viewing exercise as an obligation to considering it an essential medicine exemplifies this change: “*Pilates used to feel like an obligation, but now I know it’s the first medicine we have, so I have incorporated it*.” The gradual adaptation to physical activity, such as walking and gradually increasing the time dedicated to it, has allowed participants not only to improve their physical condition but also their emotional state: “*They have advised me a lot to go out walking… Now I manage an hour and I add Pilates, Aquagym, and every day of the week I have some activity.*”

## Discussion

The present study sought to critically examine the perceptions of patients with fibromyalgia regarding pain psychoeducation as a non-pharmacological treatment and its subsequent impact on pain magnification, self-efficacy, and biobehavioral management.

In this qualitative study utilizing IPA, we delved into the subjective experiences of fibromyalgia patients engaged in pain psychoeducation, elucidating the complexities of its influence on their pain magnification, self-efficacy, and biobehavioral management. Through the narrative lens of the patients, we aimed to dissect the multi-layered efficacy of psychoeducation and its potential to facilitate patient empowerment and adaptation.

### Understanding pain in fibromyalgia through interpretative phenomenological analysis

IPA offered a nuanced understanding of the lived experiences of individuals with fibromyalgia, echoing the sentiments expressed in previous literature that emphasize the multifaceted nature of the condition and the need for comprehensive treatment approaches (Blyth et al., 2005; Devan et al., 2018).

Moreover, IPA enabled a nuanced examination of the patients’ narratives, revealing that pain, often likened to an unwelcome yet indelible part of one’s life, requires continuous negotiation and coping strategies. This aligns with the assertions of previous research that has emphasized the chronicity and omnipresence of fibromyalgia pain and the consequent need for strategies fostering pain self-management and cognitive restructuring (Devan et al., 2018; Tomé-Pires et al., 2023).

### The psychoeducational Program’s role in pain perception and management

Patients’ portrayals of pain resonate with the metaphor of an uninvited guest, underscoring the persistent nature of their discomfort. This study extends the dialogue on pain’s persistence in fibromyalgia, affirming the complexity of its management as posited in earlier research (Nees et al., 2020).

The transition from resignation to active acceptance highlighted in patient testimonies reveals a key psychological shift facilitated by psychoeducation. However, the thin line between acceptance and learned helplessness warrants a discussion on ensuring that acceptance does not lead to disempowerment.

Interestingly, although resignation was an observable response, it was the active acceptance, possibly cultivated through psychoeducational interventions, that emerged as a pivotal step toward adaptation. This observation echoes findings from previous studies which found that active engagement in self-management predicts better outcomes in chronic pain conditions (Blyth et al., 2005; de Salvetti et al., 2012). Fibromyalgia involves a complex emotional and psychological burden, and our findings reinforce the importance of psychological interventions, as documented in a meta-analysis by Dixon et al. (2007), which highlighted their efficacy in alleviating pain.

The psychoeducation program was perceived as beneficial by the participants, with reported enhancements in functional, cognitive, and emotional capacities. These findings align with a body of research indicating the efficacy of psychoeducational interventions in improving self-efficacy and reducing pain-related disability in chronic pain populations (Wilcox et al., 2006; Mann et al., 2017).

### Shift toward active self-management

In alignment with Tomé-Pires et al. (2023), our study found that participants identified the psychoeducation program as a pivotal factor in transforming their perception and management of fibromyalgia. It provided them with tools that fostered a shift from a passive reception of care to an active, engaged approach to managing their condition.

The study underscores the importance of self-management skills. Participants described shifting from perceiving fibromyalgia as a disease to recognizing it as a manageable condition, suggesting a reconstitution of beliefs about their illness. Such shifts are important because they can lead to enhanced self-efficacy, which has been associated with improved health outcomes: Jonkman et al. (2016) posited that interventions aiming to equip patients with self-management skills can significantly enhance their ability to manage chronic conditions.

### The multifaceted impact of psychoeducation on fibromyalgia management

The discourse on disability illuminated the multifaceted impact of fibromyalgia on cognition and socio-labor participation. Patients’ experiences with cognitive impairments and socio-labor challenges showed the need for interventions that extend beyond medical treatment, advocating for a more nuanced understanding of fibromyalgia’s impact on occupational and social identity.

Patients emphasized the cognitive-functional burden and socio-occupational challenges imposed by fibromyalgia. A critical reflection on the interactions between these difficulties and existing social and occupational structures, which often fail to accommodate the needs of individuals with chronic disabilities, is essential. The analysis should address how interventions can not only enhance individual capacities but also transform the socio-occupational environment to be more inclusive.

The psychoeducation program was shown to be a pivotal element in enhancing the functional and emotional well-being of the participants. Patients reported substantial benefits in terms of cognitive-functional capacity, such as improved physical activity and better management of pain-related cognitive symptoms such as memory and concentration. Furthermore, the program’s impact on social-emotional skills was notable, with participants indicating improved emotional stability and interpersonal relationships.

Furthermore, the reported improvements in functional capacity, cognitive ability, and emotional-affective capacity suggest a benefit to psychoeducation programs (Nees et al., 2020). The program’s impact on enhancing cognitive abilities aligns with previous findings indicating that managing cognitive aspects is as important as addressing the physical symptoms of fibromyalgia (Gordon et al., 2016).

However, the limitations noted within the psychoeducation program—specifically, the demand for individualization and the expansion of topics covered—reflect concerns documented in the literature about the need for tailored interventions that address the specific needs of patients with chronic pain. Additionally, socioeconomic factors were mentioned as barriers, reinforcing the importance of external support systems for facilitating effective pain management, a concern similarly raised by Park et al. (2013) and Mann et al. (2017).

Patients also identified limitations within the psychoeducation program, particularly highlighting the need for individualized approaches and inclusion of overlooked topics such as sexual health—a domain often impacted by chronic pain yet seldom addressed in psychoeducational settings (Monga et al., 1998; Ambler et al., 2001). In addition, patients expressed a preference for having professionals specifically review exercises, due to fears of performing them incorrectly, which implies a clear need for collaboration with other healthcare professionals. This feedback shows the importance of integrating qualified therapeutic exercise professionals into the multidisciplinary team, ensuring that patients receive personalized and direct guidance on performing their exercises, mitigating their fears, and enhancing the treatment effectiveness.

In terms of fibromyalgia-related disability, the data corroborate the current literature on the profound cognitive-functional impairments and socio-labor challenges that these patients face. The study’s findings lend further credence to the call for comprehensive interventions that consider not only the biophysical aspects of the condition but also the psychosocial and occupational dimensions (Gordon et al., 2016).

The socio-labor determinants mentioned by the participants point to the broader socio-economic implications of living with a chronic condition. Policy-level interventions aimed at fostering a more inclusive and supportive work environment are necessary for individuals with disabilities.

The insights gleaned from participants regarding the Learning Elements derived from the Psychoeducation Program signify a step toward empowerment. Education in self-management techniques is a cornerstone of effective fibromyalgia management, as echoed in literature demonstrating the pivotal role of self-efficacy in pain self-management (Jensen et al., 2003; Park et al., 2013).

Our research also illuminated the value of psychoeducational interventions in empowering patients to develop pain self-management skills, a vital component of living with a chronic condition, as suggested by de Salvetti et al. (2012) and Cuperus et al. (2013). This is particularly relevant in light of the barriers to self-management identified, such as lack of motivation and physical limitations (Wilcox et al., 2006; Matthias et al., 2016).

### Limitations and future studies

Although our findings provide valuable insights into the non-pharmacological effects on quality of life and chronic pain management, several inherent limitations in the research design need to be considered.

The use of a convenience sample, composed exclusively of members of a patient association, could induce a selection bias affecting the generalizability of the results. The participants were also members of a fibromyalgia association, which might indicate a higher level of engagement and potentially better outcomes compared with the general fibromyalgia population.

Participants reported a significant enhancement in quality of life following engagement in the pain psychoeducation program. However, it is necessary to contemplate the extent to which these benefits directly result from psychoeducation and how much can be attributed to other variables, such as the inherent social support from group participation or the passage of time. Another consideration is the potential for a pleasing bias in participants’ responses, given that they might feel compelled to report anticipated improvements reflective of researcher expectations.

Participants pinpointed shortcomings in the program’s customization and suggested the inclusion of additional topics, such as sexuality and social interaction. The critique here lies in the inherent challenge of balancing the standardized structure of a psychoeducation program that benefits the collective with the need for personalization to address individual patients’ needs. Moreover, the feasibility of implementing such suggestions in a group format requires careful consideration and potentially more resources.

Future research should focus on overcoming these limitations to enrich the understanding of the studied phenomenon. Quantitative studies, such as randomized controlled trials, would be suitable for assessing the efficacy of the psychoeducation program on specific variables, such as functionality and pain management. Such studies would allow for the inclusion of objective measurements complementary to participants’ subjective reports.

It would also be relevant to investigate personalized modes of psychoeducational intervention that address individual patient needs, as well as to conduct longitudinal studies to determine the long-term effects of such programs. Additionally, the impact of socioeconomic factors on disease management and treatment response should be considered.

Detailed research offers a clear path for advancement in the understanding and treatment of fibromyalgia. A multidisciplinary approach that incorporates a broader evaluation of contextual variables and a mixed methodology could provide a more comprehensive and complete view of the efficacy of psychoeducational interventions, as well as their capacity to integrate into a comprehensive treatment regimen for fibromyalgia.

## Conclusion

In conclusion, this study demonstrates the perceived benefits of psychoeducation in managing the multifaceted challenges of fibromyalgia, highlighting its potential to improve functional, cognitive, and emotional outcomes. Nonetheless, it also identifies areas for program enhancement and the need for broader socio-economic support to optimize the benefits of such interventions. Future research should aim to explore the long-term effects of psychoeducational interventions and further examine their role as a standalone treatment modality.

## Data availability statement

The original contributions presented in this study are included in the article, further inquiries can be directed to the corresponding author.

## Ethics statement

The studies involving humans were approved by the Rey Juan Carlos University Ethical Committee. The studies were conducted in accordance with the local legislation and institutional requirements. The participants provided their written informed consent to participate in this study.

## Author contributions

SD-B: Writing – original draft, Software, Resources, Investigation, Formal analysis, Data curation, Conceptualization. RF-P: Writing – review and editing, Writing – original draft, Supervision, Project administration, Methodology, Conceptualization. JP-M: Writing – review and editing, Validation, Supervision. JF-C: Writing – review and editing, Visualization, Validation, Supervision. RL: Writing – review and editing, Visualization, Validation, Supervision, Data curation, Conceptualization.
