# Supplementary material for: Perceived benefits and limitations of a psychoeducation program for patients with fibromyalgia: an interpretative phenomenological analysis
Source: Front Psychol. 2024 Aug 14;15:1422894. doi: 10.3389/fpsyg.2024.1422894 (PMC11350163; doi:10.3389/fpsyg.2024.1422894)
Supplement: Supplementary file 3 [file Table_1.DOCX]

**Annex 1.** Interview questions.

1. How would you describe your experience with the chronic pain management education you have received?

2. Have you noticed any changes in your thoughts, attitudes and coping with pain since you started the education program?

3. What strategies or techniques learned in the education program have been most useful to you so far?

4. What would you consider to be the most important aspect of the education program?

5. What impact has the psychoeducation program had on your social and family relationships?

6. How would you describe the relationship with the therapist or educators in the education program? How important is the relationship with the educators in the learning process during education?

7. During the program have you been able to express your feelings, doubts and concerns? Has the education program been conducive to your participation?

8. Was there ever a time when you found it difficult to apply what you learned in the program to your daily life? What did you do to overcome that challenge?

9. What attributes or characteristics do you think are important for educators delivering a chronic pain program to have?

10. What suggestions would you have for improving chronic pain management education?

11. Could you provide a slogan or catch phrase that summarizes what was learned in the education program?

12. Would you recommend this education program to others living with chronic pain? Why?
